# A Birth Cohort Study of Maternal and Infant Serum PCB-153 and DDE Concentrations and Responses to Infant Tuberculosis Vaccination

**DOI:** 10.1289/ehp.1510101

**Published:** 2015-12-09

**Authors:** Todd A. Jusko, Anneclaire J. De Roos, Sue Y. Lee, Kelly Thevenet-Morrison, Stephen M. Schwartz, Marc-André Verner, Lubica Palkovicova Murinova, Beata Drobná, Anton Kočan, Anna Fabišiková, Kamil Čonka, Tomas Trnovec, Irva Hertz-Picciotto, B. Paige Lawrence

**Affiliations:** 1 Department of Public Health Sciences, and; 2 Department of Environmental Medicine, University of Rochester School of Medicine and Dentistry, Rochester, New York, USA; 3 Department of Environmental and Occupational Health, Drexel University School of Public Health, Philadelphia, Pennsylvania, USA; 4 Program in Epidemiology, Division of Public Health Sciences, Fred Hutchinson Cancer Research Center, Seattle, Washington, USA; 5 Department of Occupational and Environmental Health, School of Public Health and Université de Montréal Public Health Research Institute (IRSPUM), Université de Montréal, Montreal, Quebec, Canada; 6 Department of Environmental Medicine, and; 7 Department of Toxic Organic Pollutants, Slovak Medical University, Bratislava, Slovak Republic; 8 Research Centre for Toxic Compounds in the Environment, Masaryk University, Brno, Czech Republic; 9 Department of Analytical Chemistry, University of Vienna, Vienna, Austria; 10 Division of Environmental and Occupational Health, Department of Public Health Sciences, University of California, Davis, Davis, California, USA

## Abstract

**Background:**

Reasons for the highly variable and often poor protection conferred by the Mycobacterium bovis bacille Calmette–Guérin (BCG) vaccine are multifaceted and poorly understood.

**Objectives:**

We aimed to determine whether early-life exposure to PCBs (polychlorinated biphenyls) and DDE [1,1-dichloro-2,2-bis(p-chlorophenyl)ethylene] reduces 6-month infant BCG vaccine response.

**Methods:**

Data came from families participating in a prospective birth cohort in eastern Slovakia. At birth, maternal and cord blood were collected for chemical analyses, and infants were immunized with BCG. Blood was collected from infants for chemical analyses and to determine 6-month BCG-specific immunoglobulin (Ig) G and IgA levels. Multivariable linear regression models were fit to examine chemical–BCG associations among approximately 500 mother–infant pairs, with adjustment for confounders.

**Results:**

The median 6-month infant concentration of the prevalent congener PCB-153 was 113 ng/g lipid [interquartile range (IQR): 37–248], and 388 ng/g lipid (IQR: 115–847) for DDE. Higher 6-month infant concentrations of PCB-153 and DDE were strongly associated with lower 6-month BCG-specific antibody levels. For instance, BCG-specific IgG levels were 37% lower for infants with PCB-153 concentrations at the 75th percentile compared to the 25th percentile (95% CI: –42, –32; p < 0.001). Results were similar in magnitude and precision for DDE. There was also evidence of PCB–DDE additivity, where exposure to both compounds reduced anti-BCG levels more than exposure to either compound alone.

**Conclusions:**

The associations observed in this study indicate that environmental exposures may be overlooked contributors to poorer responses to BCG vaccine. The overall association between these exposures and tuberculosis incidence is unknown.

**Citation:**

Jusko TA, De Roos AJ, Lee SY, Thevenet-Morrison K, Schwartz SM, Verner MA, Palkovicova Murinova L, Drobná B, Kočan A, Fabišiková A, Čonka K, Trnovec T, Hertz-Picciotto I, Lawrence BP. 2016. A birth cohort study of maternal and infant serum PCB-153 and DDE concentrations and responses to infant tuberculosis vaccination. Environ Health Perspect 124:813–821; http://dx.doi.org/10.1289/ehp.1510101

## Introduction

Tuberculosis is a major global public health problem, ranked in 2013 as the 11th leading cause of years of life lost globally (GBD 2013 Mortality and Causes of Death Collaborators 2014). One strategy for reducing the incidence of tuberculosis has been through *Mycobacterium bovis* bacille Calmette–Guérin (BCG) vaccination. BCG is a live, attenuated vaccine typically administered around the time of birth. The BCG vaccine substantially reduces the risk of disseminated, severe forms of tuberculosis in early childhood (Trunz et al. 2006), but the protection that early-life immunization confers against pulmonary tuberculosis in older children and adults is variable and generally poor (Corbel et al. 2004; Fine 1995). The reasons for the highly variable and less than ideal effectiveness of the BCG vaccine are not known, and have been debated, sometimes quite hotly, for many years (Abebe 2012). However, generally overlooked in this discussion are the potential contributions of early-life exposures to the initial host response to BCG immunization or to the maintenance of immune protection it affords over time.

Recent evidence suggests that early-life chemical exposures alter the developing immune system, including decreased antibody responses to some vaccines. For example, higher polychlorinated biphenyl (PCBs) and perfluoroalkyl substance (PFAS) serum concentrations have been associated with lower responses to tetanus and diphtheria vaccines during childhood (Grandjean et al. 2012; Heilmann et al. 2006), and higher maternal and infant PCB concentrations have been associated with a reduced volume of the infant thymus, the site of T-cell maturation (Jusko et al. 2012). Development of the immune system is a complex and intricate process, requiring coordinated events that span gestation through early postnatal life. Even small changes to these events may lead to long-term alterations to immune function. Support for this idea comes from animal models that demonstrate that exposure to 2,3,7,8-tetrachlorodibenzo-*p*-dioxin (TCDD) during gestation and shortly after birth results in functional changes that persist into adult life (Gehrs and Smialowicz 1999; Sugita-Konishi et al. 2003; Vorderstrasse et al. 2004). Changes reported include alterations to the immune cells that control antibody production and the formation of immunological memory, a fundamental tenet of vaccination strategies. Taken together, these studies suggest that chemical exposures occurring during early life may increase the susceptibility to vaccine-preventable diseases in adulthood. Some authors have suggested that environmental toxicants may be a “missing link” in the global battle against infectious disease (Winans et al. 2011).

In the present study we focused on two chemicals of concern, PCBs and DDE [1,1-dichloro-2,2-bis(*p*-chlorophenyl)ethylene]. Both organochlorines are considered persistent organic pollutants (POPs) that resist environmental and biological degradation, are lipophilic, and bioaccumulate in human tissues (ATSDR 2000, 2002). PCBs were once used in commercial and consumer applications because of their desirable physical and chemical properties (e.g., water insolubility, low flammability, and chemical stability) (ATSDR 2000). PCB levels have declined in human tissues in many parts of the world (Fängström et al. 2005). Yet there is still concern about their potential health effects, particularly among populations living in areas with significant environmental contamination (Jusko et al. 2014; Pavuk et al. 2014) or those whose diet includes heavy consumption of PCB contaminated seafood (Ayotte et al. 2005; Fängström et al. 2005). DDE is the primary metabolite of DDT, an insecticide, which is still used in areas of the world where malaria vector control is needed (van den Berg 2009). Both DDE and PCBs can cross the placenta and mammary glands, and therefore can be readily passed from mother to infant (Al-Saleh et al. 2012; Glynn et al. 2011; Lancz et al. 2015; Needham et al. 2011). To determine whether exposure to these POPs is associated with decreases in infant BCG-specific antibody levels, we conducted an investigation among a cohort of mother–infant pairs recruited from two districts in eastern Slovakia.

## Methods

### Study Population and Sample Selection

Mother–infant pairs participating in the present study were enrolled from two districts in eastern Slovakia: Michalovce, an area with substantial environmental PCB contamination (Kocan et al. 2001), and Svidnik, an area located approximately 70 km to the northwest. Pollution in the Michalovce region results, in part, from a chemical-manufacturing facility that produced PCBs between 1959 and 1984. Women were approached to participate in our study between 2002 and 2004 at the time they came to the local hospital in either Michalovce or Svidnik to deliver their child. At that time, mothers gave informed consent to participate. Because each district has only one hospital, the vast majority of women giving birth during this period delivered at either hospital. We excluded *a*) mothers with more than four previous births, *b*) mothers < 18 years of age, *c*) mothers who had resided < 5 years in their district, *d*) mothers with a major illness during pregnancy, and, after delivery, *e*) mothers of infants who had severe birth defects. In total, 1,134 women were enrolled in both districts (811 in Michalovce and 323 in Svidnik). Mother–infant pairs were followed up by study staff for 6 months after the child’s birth, at which time 973 mother–infant pairs were still participating (86%). The study protocol was approved by institutional review boards at the University of California at Davis and the Slovak Medical University.

### Blood Collection

Maternal blood was drawn during the mother’s delivery hospital stay, and cord blood was collected just after delivery. At the 6-month visit, up to 9 mL of blood was collected from the infant. Details on the handling of specimens and isolation of serum have been presented elsewhere (Jusko et al. 2010). From the isolated 6-month infant serum, approximately 500 μL was aliquoted into each microtube (up to two tubes total) and stored frozen at –20°C. Of the 973 mother–infant pairs participating at 6-months, 960 infants (99%) received a 6-month blood draw, and a sufficient volume of serum for anti-BCG analysis was available for 541.

### Chemical Measurement

Maternal, cord, and 6-month infant chemical concentrations were determined at the Department of Toxic Organic Pollutants at the Slovak Medical University in Bratislava using high-resolution gas chromatography with electron capture detection (HP 5890; Hewlett-Packard). This laboratory serves as the National Reference Laboratory for Dioxins and Related Compounds for the Slovak Republic and has been certified by the Slovak National Accreditation Service (ISO/IEC 17025:2005, certification no. S-111). Further, the laboratory participates in interlaboratory comparison tests, such as the Interlaboratory Quality Assessment coordinated by the World Health Organization (G-EQUAS 2009; WHO 2000). The concentrations of 15 PCB congeners [International Union of Pure and Applied Chemistry (IUPAC) numbers 28, 52, 101, 105, 114, 118, 123+149, 138+163, 153, 156+171, 157, 167, 170, 180, and 189] and *p,p*´DDE were determined using a process which involved solid-phase extraction, cleanup, and quantitation (Conka et al. 2005; Kocan et al. 1994). Quantification was based on the calibration curve generated by authentic PCB and DDE standard solutions at five different concentration levels. Quality control activities consisted of analyses of samples in batches of 10 run simultaneously with a blank sample and in-house reference material (spiked porcine serum); response for a particular congener had to be in the range of 90–110% using the concentration of the middle point of the calibration curves for that congener. We calculated the limit of detection (LOD) for each analyte by considering the ratio of background to noise (multiplied by three) and the peak height of the analyte in standard solution of the lowest concentration. For a portion of 6-month samples (29%), high-resolution mass spectrometry (HRMS) (Chovancová et al. 2012) was used to quantitate PCB concentrations instead of high-resolution gas chromatography with electron capture detection, as these samples were analyzed subsequently with newer technology. The same laboratory performed both analytic procedures.

### Lipid Measurement

Lipid concentrations were estimated by the enzymatic summation method (Akins et al. 1989). Total serum cholesterol (TC) and triglyceride (TG) concentrations were determined at the Department of Clinical Biochemistry of TOP-MED General Hospital Bratislava using a DuPont Automatic Clinical Analyzer III, and cholesterol oxidase without cholesterol esterase was used to detect free cholesterol (FC). The method of Takayama et al. (1977) was used to determine serum choline–containing phospholipids (PL). Total serum lipids were calculated using the formula: TL = 1.677 × (TC – FC) + FC + TG + PL.

### BCG Vaccination

During the study period in Slovakia, a mandatory BCG vaccination was given approximately 4 days after birth. The live, attenuated vaccine is given intradermally when mother and neonate are still in the hospital. According to the WHO and the United Nations Children’s Fund, 98% of all Slovak infants received this vaccination in the years 2002–2004 (the years that children in our study were born) (WHO 2006). Infants in our cohort were vaccinated with one of three different BCG vaccines: *a*) BCG Behringer, strain Kopenhagen 1331; *b*) Lyophilized BCG, strain Mérieux (Glaxo/Sanofi) 1077; or *c*) BCG Statens Serum Institut, Danish strain 1331, as all three vaccines were in use in the Slovak Republic from 2002 to 2004 (Otrusinik R, personal communication). After August 2004, BCG SSI was used exclusively.

### BCG-Specific Antibody Enzyme-Linked Immunosorbant Assay

BCG-specific antibody levels in serially diluted 6-month infant serum samples were determined by stacking antibody isotype-specific enzyme-linked immunoassay (ELISA), as previously described (Boule et al. 2014). BCG was purchased from Sanofi Pasteur and reconstituted and diluted as per manufacturer instructions. Coating antigen was prepared by sonication and heat inactivation (Shirtcliffe et al. 2004). Because we did not know which BCG vaccine formulation infants received, we piloted the ELISA using both Sanofi and SSI strains (the two main strains that infants were vaccinated with). A comparison of results for the two strains revealed that absorbance values were strongly correlated for both immunoglobulin (Ig) G and IgA (*r* > 0.9), suggesting that the measured anti-BCG levels would not be strain dependent. Because the Sanofi strain produced slightly higher absorbance values (data not shown), it was chosen as the coating antigen for analyses. Ninety-six–well plates were coated with BCG at 0.1 μg/mL for IgG isotype measurements and 0.5 μg/mL for the IgA isotype. Six-month infant serum samples were prepared in a series of 3-fold dilutions ranging from 1:25 to 1:6,075. A serially diluted reference sample was used on all plates. Biotinylated goat anti-human IgG or IgA antibodies (Southern Biotech) were used to detect and compare the relative amount of each antibody isotype.

### Questionnaires and Medical Records

After enrollment at the time of delivery, women were administered a questionnaire by trained staff which elicited information about maternal health, past pregnancies, tobacco use, family living environment, and sociodemographic information. No women reported tuberculosis infection during pregnancy. The lack of maternal or infant tuberculosis infection through age 6 months was confirmed by the study physician. Women were considered smokers if they reported smoking during pregnancy or stated that they were a current smoker at the delivery interview. Romani ethnicity was assigned if the ethnic origin of either of the mother’s parents was Romani, the Romani language was spoken at home, or the mother was planning to raise her child with the Romani language. Otherwise, ethnicity was assigned as Slovak/other European. From the newborn medical record, we abstracted information about the perinatal health of the infant and the child’s birth weight and gestation length. Gestation length was based on the date of the woman’s last menstrual period and the judgment of her physician. An additional questionnaire was administered during the infant’s 6-month visit, which took place at either hospital in the Department of Pediatrics. The questionnaire included questions about the home environment of the child and breastfeeding habits. Mothers were asked whether they were presently breastfeeding, and if not, when they stopped. They were also asked if and when they began using a milk substitute.

### Pharmacokinetic Modeling of Postnatal PCB and DDE Concentrations

To estimate additional PCB-153 and *p,p´*-DDE exposure parameters beyond the measured concentrations, we used a previously validated physiologically based pharmacokinetic (PBPK) model (Verner et al. 2013) that incorporates information on the mother’s age at delivery, prepregnancy body weight, gestational weight gain, infant’s weight at birth and at 6 months, gestational age at birth, the duration of breastfeeding and exclusivity, infant’s sex, maternal and cord plasma concentrations, and the half-lives of PCB-153 and *p,p´*-DDE. For each child in the study, we estimated monthly postnatal concentrations from 1 to 6 months, the cumulative exposure during the first 6 months [i.e., the area under the concentration × time curve (AUC)], and the peak concentration reached during that period. We also estimated peak and AUC measures for the entire pre- and postnatal period.

### Data Analysis

***PCB and DDE concentration.*** For the present analysis, we used PCB-153 (hereafter, PCB) as a surrogate for the 15 total PCB congeners measured in serum. This was done for two reasons: *a*) PCB-153 is highly correlated with total PCB concentration in this cohort (ρ > 0.99 for maternal, cord, and 6-month exposures), and *b*) PCB-153 was detectable in all maternal and infant specimens. For the < 1% of *p,p´*-DDE (hereafter, DDE) concentrations that were below a limit of detection, we used the values as reported by the laboratory (i.e., no substitution or imputation). Two maternal, three cord, and two DDE measures were below a limit of detection.

***Statistical models.*** The outcome variables—BCG-specific IgG and IgA levels—were modeled as the absorbance value recorded from the ELISA plate. From the 3-fold dilutions ranging from 1:25 to 1:6,075, we modeled the 1:225 dilution because this was in the middle of the linear range of the dilution curve (see Figures S1–S3). Absorbance values were transformed by the natural log to normalize residuals.

To select potential confounding variables, we created a directed acyclic graph (DAG) (Greenland et al. 1999) to identify a minimally sufficient set of adjustment variables to reduce confounding. We regarded the causal structure of the maternal and cord exposure models to be identical, and adjusted these models for ethnicity (Slovak/other European vs. Romani), maternal smoking (yes/no), education (< 12, 12–16, or ≥ 17 years), parity (0–4), and maternal age at the child’s birth (years). Six-month exposure models included adjustment for ethnicity, maternal age, and maternal education (DAGs not shown). Because the role of breastfeeding as a confounder of the postnatal PCB/DDE–anti-BCG association can be debated, 6-month exposure models are presented both with and without adjustment for the duration of exclusive breastfeeding (months). For the PBPK models, postnatal only models were adjusted for the same set of potential confounders as the 6-month models above, except that exclusive breastfeeding duration was truncated at the exposure time point of interest (e.g., the duration was truncated at 3 months in the 3-month PBPK regression model). PBPK models that included both the pre- and postnatal period used the same covariate set as the maternal/cord exposure models, and results are shown both with and without adjustment for exclusive breastfeeding duration. Chemical concentrations were modeled as natural log transformed concentrations on a per lipid basis (nanograms per gram lipid), and estimated associations are reported as the percent difference in the relative absorbance level for an interquartile range difference in exposure. The MIXED procedure in SAS was used to estimate each chemical–BCG level association and associated 95% confidence interval and *p*-value (version 9.4; SAS Institute Inc.). In light of research on endocrine-disrupting compounds and observations of sexually dimorphic findings in terms of neurodevelopment and other developmental processes, we initially examined measured chemical concentrations and anti-BCG levels by stratifying on infant sex, but found little evidence of heterogeneity (data not shown), and therefore report chemical–anti-BCG associations without sex stratification.

First, we examined individual associations between maternal, cord, and 6-month infant PCB concentrations and BCG-specific IgG and IgA. Identical models then addressed maternal, cord, and 6-month infant DDE in relation to BCG-specific IgG and IgA. Then, to assess the unique contributions of PCB and DDE, several approaches were taken. First, because PCB and DDE concentrations were only moderately correlated for the maternal (ρ = 0.54; *p* < 0.0001) and cord measures (ρ = 0.55; *p* < 0.0001), we included models with simultaneous inclusion of both exposures. A second approach was used for the 6-month chemical–BCG associations, owing to the strong correlation between PCB and DDE (ρ = 0.86). We dichotomized each chemical exposure at the median into “high” and “low” exposure categories. Anti-BCG antibody levels were then modeled as a function of dummy-coded PCB and DDE variables and the interaction between the two (i.e., main effects for both PCB and DDE, and the interaction term). This parameterization allowed the estimation of, and contrasts between, the four categories of exposure: *a*) low PCB and low DDE; *b*) low PCB and high DDE; *c*) high PCB and low DDE; and *d*) high PCB and high DDE, and permitted us to statistically evaluate the presence or absence of exposure additivity between 6-month PCB and DDE concentrations.

***Sensitivity analyses.*** We conducted a series of sensitivity analyses. These models were identical to our primary 6-month infant PCB/DDE and BCG-specific IgG model, except that *a*) PCB/DDE concentration was entered into the regression model on a wet-weight basis (nanograms per milliliter) and 6-month infant total lipid concentration was included as a separate covariate (Schisterman et al. 2005); *b*) we included additional covariates that either did not meet the criteria for our DAG but have been reported in the literature as potential confounders of the relation between PCB/DDE and immune function or may be strong predictors of the outcome [maternal smoking, parity, district of residence (Michalovce or Svidnik), and child age at 6-month blood draw (essentially “time since vaccination”)]; *c*) we added a categorical variable for the BCG ELISA batch date; *d*) we added an indicator variable to the primary model for high-resolution gas chromatography with electron capture detection versus HRMS, to confirm that the 29% of 6-month infant serum samples analyzed by HRMS did not bias our results; *e*) we removed the top and bottom 3% of PCB/DDE concentrations to evaluate the influence of extreme values; and *f*) we combined all the previous sensitivity model specifications to evaluate the total effect of these alternative specifications.

## Results

### Study Population

Table 1 shows characteristics of the 541 mother–infant pairs who had adequate 6-month serum for the measurement of BCG antibody levels, the complete group of those followed-up at 6 months (*n* = 973), and the full cohort (*n* = 1,134). Overall, these subsets of the cohort were very similar. Among the 541 mother–infant pairs in the present study, 50% of infants were female, 1% were born preterm (< 37 weeks of gestation), and 5% of infants were born low birth weight (< 2,500 g). The majority (81%) of infants were of Slovak or other European ethnicity, and 19% were Romani. Of the 541 mother–infant pairs with adequate serum for the measurement of BCG antibody levels, complete data for the multivariable models (discussed below) were available for 480 mother–infant pairs in maternal exposure models, 492 in models that used cord blood concentrations, and 454 in models where 6-month infant concentrations were the exposure of interest. For the PBPK models, up to 517 mother–infant pairs were included.

**Table 1:** Characteristics of infants and mothers in the full cohort, those with follow-up at 6 months, and those available for BCG analysis [*n* (%)].*^a^*

Characteristic	BCG study (*n *= 541)	6-month follow-up (*n *= 973)	Full cohort (*n *= 1,134)
Infant sex
Female	269 (50)	480 (49)	547 (48)
Male	272 (50)	493 (51)	587 (52)
District of residence
Michalovce	479 (89)	710 (73)	811 (72)
Svidnik	62 (11)	263 (27)	323 (28)
Gestation length (weeks)
< 37	7 (1)	22 (2)	31 (3)
37–41	510 (95)	912 (95)	1,059 (94)
≥ 42	19 (4)	29 (3)	32 (3)
Missing^*b*^	5	10	12
Birth weight (g)
< 2,500	25 (5)	41 (4)	50 (4)
2,500–3,499	301 (56)	550 (57)	645 (57)
≥ 3,500	215 (40)	380 (39)	435 (38)
Missing	0	2	4
Parity
0	225 (42)	403 (42)	471 (42)
1	190 (35)	322 (33)	371 (33)
2	84 (16)	169 (17)	196 (17)
3	30 (6)	61 (6)	76 (7)
4	10 (2)	16 (2)	17 (2)
Missing	2	2	3
Maternal age at child’s birth (years)
18–< 20	52 (10)	84 (9)	95 (8)
20–30	403 (74)	717 (74)	850 (75)
≥ 31	86 (16)	172 (18)	189 (17)
Ethnicity of child
Romani	103 (19)	190 (20)	236 (21)
Slovak/other European	438 (81)	783 (80)	898 (79)
Marital status
Married	475 (91)	878 (93)	1,026 (93)
Never married	44 (8)	61 (6)	69 (6)
Divorced/separated	5 (1)	7 (1)	9 (1)
Missing	17	27	30
Maternal education (years)
< 12	210 (40)	366 (39)	441 (40)
12–16	282 (54)	527 (56)	598 (54)
> 16	33 (6)	53 (6)	65 (6)
Missing	16	27	30
Exclusive breastfeeding (months)
0	18 (4)	33 (4)	—
> 0–1	87 (17)	126 (14)	—
> 1–3	129 (25)	222 (25)	—
> 3–< 6	63 (12)	109 (12)	—
6	212 (42)	403 (45)	—
Missing	32	80	—
Maternal smoking during pregnancy
Yes	146 (28)	233 (25)	233 (25)
No	382 (72)	694 (75)	694 (75)
Missing	13	46	207
^***a***^Percents may not sum to 100 because of rounding. ^***b***^Missing values not included in percentages.			

### PCB and DDE Concentrations

PCB and DDE concentrations were detected in > 99% of maternal and infant samples (Table 2). PCB concentrations were highest in maternal serum (median, 150 ng/g lipid), compared with cord (median, 115 ng/g lipid) and 6-month infant serum (median, 113 ng/g lipid); DDE showed a similar pattern of median lipid-basis concentration over time (Table 2). PCB and DDE concentrations had higher extreme values at 6 months compared with maternal or cord concentrations. The cross-sectional correlation between PCB and DDE concentrations on a wet-weight basis was strongest at 6 months (ρ = 0.86; *p* < 0.0001) compared with cord (ρ = 0.55; *p* < 0.0001) and maternal serum (ρ = 0.54; *p* < 0.0001) correlations. Maternal and cord PCB concentrations were predictive of 6-month infant PCB concentrations (0.37 ≤ ρ ≤ 0.38; *p* < 0.0001) and maternal and cord DDE concentrations were predictive of 6-month infant PCB concentrations (0.42 ≤ ρ ≤ 0.45; *p* < 0.0001). The correlation between duration of exclusive breastfeeding and 6-month PCB was ρ = 0.63 (*p* < 0.0001), and the correlation between duration of exclusive breastfeeding and 6-month DDE was ρ = 0.63 (*p* < 0.0001).

**Table 2:** Chemical concentrations in serum among participants with complete model data.*^a^*

Exposure	*n*	*n* > LOD (%)	Mean	Minimum	P25	P50	P75	Maximum
PCB-153
Wet weight (ng/mL serum)
Maternal	480	480 (100)	2.1	0.2	1.0	1.5	2.4	56.1
Cord	492	492 (100)	0.4	0.0	0.2	0.3	0.5	6.3
6-month infant	454	454 (100)	1.3	0.0	0.2	0.7	1.5	33.2
Lipid basis (ng/g lipid)
Maternal	480		205	15	102	150	234	3,958
Cord	492		167	2	73	115	188	3,989
6-month infant	454		206	3	37	113	248	4,714
*p,p’*-DDE
Wet weight (ng/mL serum)
Maternal	480	478 (99.6)	6.0	0.0	2.6	4.6	7.6	39.8
Cord	492	489 (99.4)	1.4	0.0	0.6	1.1	1.8	9.1
6-month infant	454	452 (99.6)	4.1	0.0	0.7	2.4	5.4	46.0
Lipid basis (ng/g lipid)
Maternal	480		578	0.2	265	458	725	3,292
Cord	492		572	2	259	442	707	4,396
6-month infant	454		648	0.2	115	388	847	6,588
P, percentile. ^***a***^Statistics computed using lab-reported values for those below the limit of detection (LOD).								

### Measured PCB and DDE Concentrations in Relation to 6-Month Anti-BCG Levels

**Figure 1:**
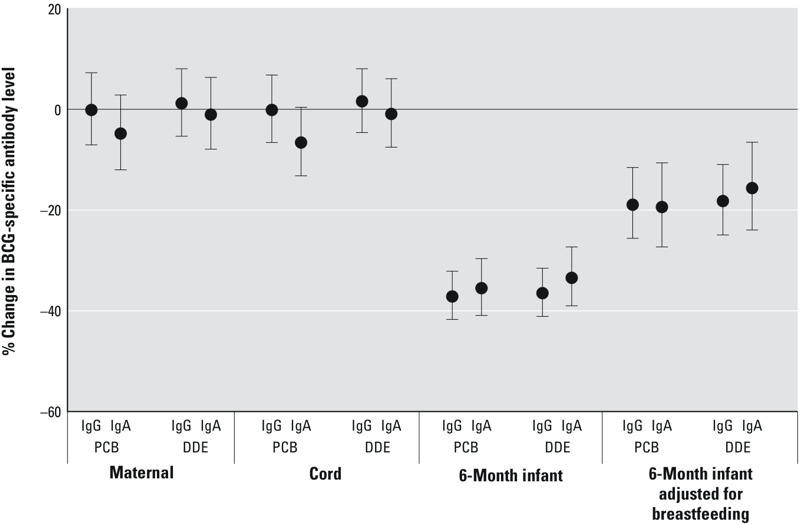
Measured maternal, cord, and 6-month infant serum PCB and DDE concentrations and the percent change (95% CI) in 6-month BCG-specific IgG and IgA levels for an interquartile range difference in each exposure. Maternal and cord serum models are adjusted for maternal ethnicity, education, age, smoking, and parity. Six-month infant models are adjusted for maternal ethnicity, education, and age. A second 6-month model includes maternal ethnicity, education, age, and the duration of exclusive breastfeeding. Interquartile ranges as well as crude and adjusted numerical estimates are available in Table S1.

Maternal (*n* = 480) and cord (*n* = 492) serum PCB and DDE concentrations showed little evidence of dose response with IgG-specific anti-BCG levels in adjusted models, though there was some evidence of an association between cord PCB concentrations and BCG-specific IgA levels (Figure 1; see also Table S1 for regression results). The inclusion of both chemicals in maternal and cord models did not appreciably change the estimated associations. In contrast, higher 6-month infant PCB and DDE concentrations were associated with lower BCG-specific antibody levels, after adjusting for maternal ethnicity, education, and age. BCG-specific IgG levels were 37% lower, on average, for participants with PCB concentrations at the 75th percentile compared to the 25th percentile [95% confidence interval (CI): –42, –32; *p* < 0.001], and estimates for DDE were very similar in magnitude and precision. The estimated associations for both 6-month PCB and DDE were attenuated by approximately 50% after additional adjustment for duration of exclusive breastfeeding. The dose response across quartiles of 6-month serum PCB and DDE concentration was apparent regardless of the serum dilution (see Figure S3).

In the 6-month infant serum PCB and DDE multivariable models (*n* = 454), Romani infants had BCG-specific IgG levels approximately 30% lower than Slovak/other European infants. When duration of exclusive breastfeeding was included as a covariate, each month of exclusive breastfeeding was associated with a 12% lower BCG-specific IgG level; other covariates were not meaningfully predictive of BCG-specific IgG level (data not shown).

### PBPK Models and Anti-BCG Levels

**Figure 2:**
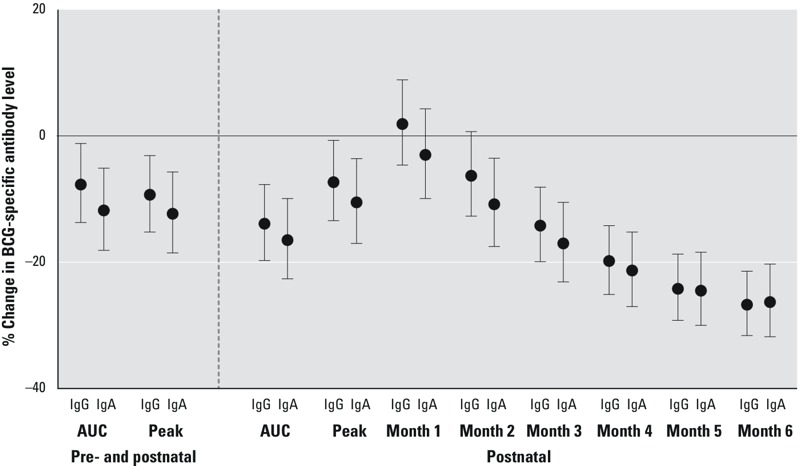
Estimated PCB concentrations from the physiologically based pharmacokinetic model (PBPK) and the percent change (95% CI) in 6-month BCG-specific IgG and IgA levels for an interquartile range difference in each PCB exposure. Pre- and postnatal models are adjusted for maternal ethnicity, education, age, smoking, and parity, and postnatal models are adjusted for maternal ethnicity, education, and age. Interquartile ranges as well as crude and adjusted numerical estimates are available in Table S2.

***PCB.*** AUC PCB models that included both the pre- and postnatal period showed evidence of an inverse association with IgG- and IgA-specific BCG antibody levels, though the association was slightly stronger for IgA-specific antibody levels (Figure 2; see also Table S2 for regression results). Peak PCB concentration during the pre- and postnatal period was also associated with lower BCG-specific antibody levels. Postnatal month–specific results were consistent with findings from the measured maternal, cord, and 6-month infant analyses: PCB measurements closest to birth were not associated with anti-BCG antibody level, whereas estimates of exposure closest to 6 months of age were strongly and inversely associated. The postnatal AUC measure of PCB exposure, as estimated from the PBPK model, was strongly and inversely associated with antibody levels; the peak exposure variable was attenuated compared to the AUC measure (despite the peak exposure having a larger interquartile range: peak AUC IQR = 240, postnatal AUC IQR = 195; see also Table S2). In this population, an IQR difference in postnatal AUC PCB exposure was associated with a 14% lower BCG-specific IgG level (95% CI: –20, –8) (see Table S2). As with the measured 6-month infant serum PCB concentrations, results from the PBPK models were attenuated after adjustment for duration of exclusive breastfeeding (see Table S2).

**Figure 3:**
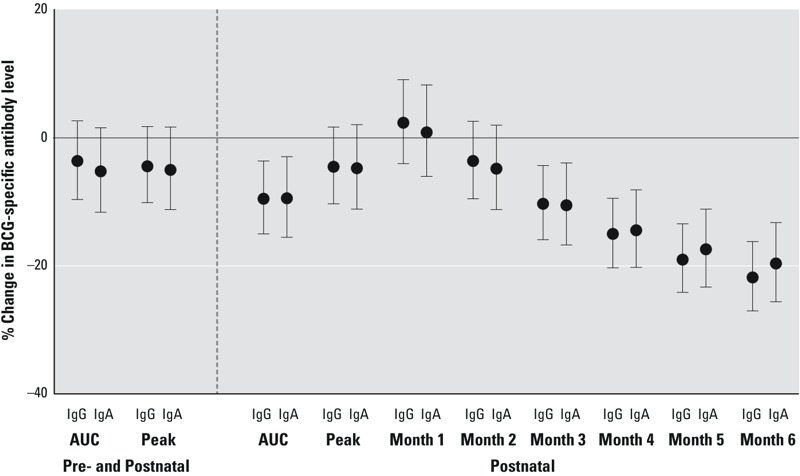
Estimated DDE concentrations from the physiologically based pharmacokinetic model (PBPK) and the percent change (95% CI) in 6-month BCG-specific IgG and IgA levels for an interquartile range difference in each DDE exposure. Pre- and postnatal models are adjusted for maternal ethnicity, education, age, smoking, and parity, and postnatal models are adjusted for maternal ethnicity, education, and age. Interquartile ranges as well as crude and adjusted numerical estimates are available in Table S3.

***DDE.*** Compared with PCB, AUC and peak DDE concentrations for the pre- and postnatal period were not as strongly associated with BCG-specific antibody levels (Figure 3; see also Table S3). Like PCB, however, month-specific DDE concentrations more proximal to 6 months were more strongly associated with lower BCG-specific antibody levels, and postnatal AUC measures of DDE exposure were inversely associated with BCG-specific antibody levels. Adjustment for duration of exclusive breastfeeding again attenuated most associations.

### PCB, DDE, and Anti-BCG Antibody Levels

**Figure 4:**
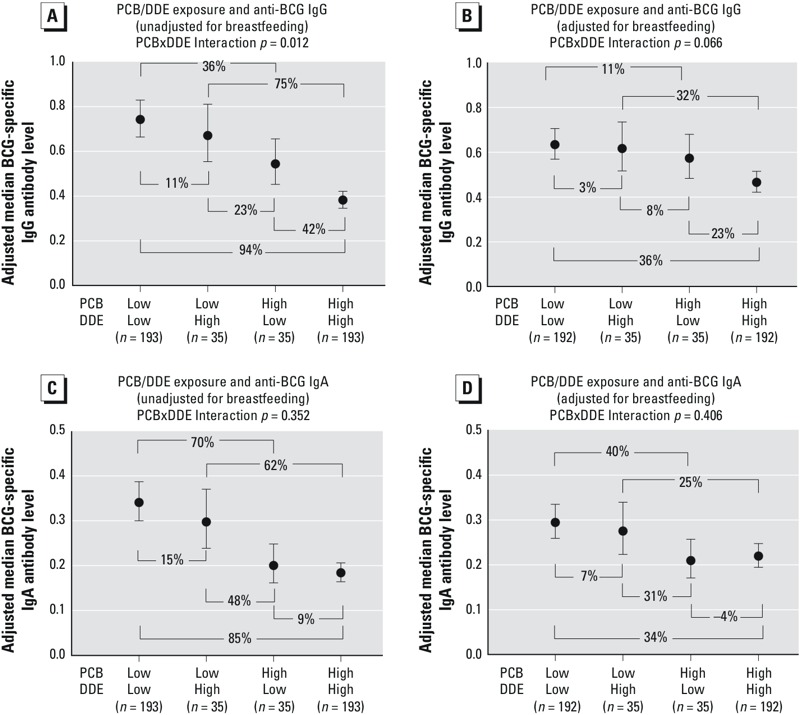
Six-month infant serum PCB and DDE concentrations, categorized as above (“high”) and/or below (“low”) the median value, in relation to 6-month BCG-specific IgG (*A,B*) and IgA (*C,D*). Results are adjusted for maternal ethnicity, education, and age (*A,C*) and maternal ethnicity, education, age, and duration of exclusive breastfeeding (*B,D*). Adjusted numerical estimates are available in Table S4.

Figure 4 shows the association between BCG-specific antibody levels and 6-month concentrations of PCB and DDE, categorizing PCB and DDE exposure as above or below the median (see also Table S4). For both IgG and IgA isotypes, a clear dose response was evident: Infants with PCB and DDE concentrations above the median (“high/high”) had the lowest antibody levels, whereas infants who were below the median for PCB and DDE (“low/low”) had higher antibody levels (above median PCB and DDE group vs. below median PCB and DDE group: –94%; 95% CI: –116, –74). Concentrations above the median of either PCB or DDE but not the other showed BCG levels intermediate to the low/low and high/high groups. The contrast between high PCB/low DDE and low PCB/low DDE tended to be greater than the contrast between high DDE/low PCB and low PCB/low DDE regardless of antibody isotype. In both IgG and IgA models, adjustment for breastfeeding tended to attenuate the differences between exposure categories. Evidence of joint effects for PCB and DDE was also observed: For IgA-specific anti-BCG levels, exposure to both compounds appeared to be additive, and for IgG-specific anti-BCG, more than additive. For example, IgA-specific anti-BCG levels were 85% lower in the high PCB/high DDE group relative to the low PCB/low DDE group, compared with 70% lower in the high PCB/low DDE group and 15% lower in the low PCB/high DDE group, relative to the low PCB/low DDE group. For IgG, anti-BCG levels were 94% lower in the high PCB/high DDE group relative to the low PCB/low DDE group, compared with 36% lower in the high PCB/low DDE group and 11% lower in the low PCB/high DDE group, relative to the low PCB/low DDE group, suggesting more than additive joint effects. Consistent with the estimated contrasts above, there was a statistical interaction between 6-month PCB and DDE concentration on IgG-specific BCG levels, both in models unadjusted for breastfeeding duration (*p* = 0.012) and adjusted for breastfeeding duration (*p* = 0.066), but there was little evidence for statistical PCB × DDE interaction in IgA-specific models.

### Sensitivity Analyses

Overall, results using different model specifications or truncating the PCB/DDE concentrations did not meaningfully differ from our primary model (see Figure S4).

## Discussion

The results from this prospective birth cohort study suggest that two common environmental contaminants, PCB-153 and *p,p´-*DDE, substantially reduce the infant response to BCG vaccination. Six-month infant PCB and DDE concentrations were strongly and inversely associated with BCG-specific IgG and IgA levels measured at 6 months, and the estimated associations were precise. The results from models using estimated concentrations from validated PBPK models were in accordance with results from measured chemical concentrations in serum, suggesting that postnatal exposures more proximal to the outcome are most strongly associated with lower anti-BCG antibody levels. Finally, we observed evidence of exposure additivity, where infants with high exposures to both compounds exhibited lower BCG-specific antibody levels compared to infants with high concentrations of either compound.

In this study, higher measured postnatal concentrations of PCBs and DDE were associated with lower antibody levels, but measured perinatal concentrations (maternal and cord) were not. Interestingly, a similar finding was observed in this cohort in relation to PCBs with thymus volume, also measured at 6 months of age in the infant. In that study, where thymus volume was estimated using ultrasound, 6-month total PCB concentration was associated with a 0.4–standard deviation decrease in thymus volume, whereas total maternal PCB concentration was not associated with altered thymus volume (Jusko et al. 2012). Evidence of the post- versus prenatal association has also been observed in other cohort studies. For instance, in a study of mother–child pairs highly exposed to PCBs in the Faroe Islands, PCB concentrations were determined in maternal and child serum, and antibody levels were measured at 18 months of age (Heilmann et al. 2006). Results from that study showed that higher postnatal rather than prenatal PCB concentrations were associated with lower 18-month diphtheria and tetanus toxoid levels. The responses to diphtheria and tetanus toxoid vaccination were also assessed in the Faroese cohort at age 5 and 7 years. At the later childhood examination, 18-month child PCB concentrations were most predictive of vaccine response, compared to prepregnancy, milk, or concurrent PCB concentrations (Heilmann et al. 2010). Experimental studies in rodents also show that early postnatal exposure, via mothers’ milk, significantly perturbed adaptive immune responses in adult offspring, whereas developmental exposure solely during gestation had a less pronounced effect (Hogaboam et al. 2008). Given that the immune system starts to develop *in utero* but continues to develop after birth, the results of the present study and others suggest that the early postnatal period is a critical period for immune system development and for its particular sensitivity to perturbation by environmental agents.

In addition to the measured chemical concentrations in maternal, cord, and 6-month infant serum, the use of a validated PBPK model permitted estimation of exposure metrics during the postnatal period (AUC, peak, and month-specific concentrations), as well as metrics spanning both the gestational and the postnatal period (AUC and peak). Overall, the estimated inverse association between chemical concentration and BCG-specific antibody levels from the PBPK models tended to be stronger for PCB than DDE. There was also evidence that the PCB–antibody association was stronger than the DDE–antibody association when both exposures were dichotomized and modeled simultaneously. At the same time, there was a suggestion that, for infants with both PCB and DDE above the median, the decrease in IgG-specific BCG antibody levels was greater than expected based on the decreases in IgG associated with high PCB alone and high DDE alone. Taken as a whole, these results suggest that *a*) PCBs exert a greater influence on anti-BCG antibody levels than does DDE, and *b*) that the combination of both exposures may more adversely affect anti-BCG antibody levels, compared with either exposure individually. A strength of using PBPK models in addition to our measured concentrations is that we were able to estimate peak concentrations spanning the postnatal as well as the combined pre- and postnatal period. Interestingly, we observed stronger associations with estimated 6-month infant concentrations compared with peak concentrations. This finding suggests that the association between PCB and DDE and anti-BCG level is time- and dose-specific, rather than only dose-specific. This provides further evidence that the early postnatal period may be a critical period during which environmental exposures perturb the developing immune system.

These analyses also provide intriguing evidence to support that the chemical–antibody associations differed by antibody isotype, particularly for PCB exposure. For instance, there was some suggestion that higher cord PCB concentration was associated with lower IgA- but not IgG-specific BCG antibody levels. This was also evidenced in the PBPK models examining AUC and peak concentrations (pre- and postnatal). Furthermore, when examining the combined exposure of PCB and DDE, IgA was less strongly associated with DDE, compared with PCB. For instance, the BCG-specific antibody level in the high PCB/high DDE group was only 9% lower than the high PCB/low DDE group for the IgA isotype (Figure 4C), but 42% lower comparing those same groups for IgG-specific BCG (Figure 4A). Thus, there may be differences in the association between PCB and DDE exposure and anti-BCG levels for different antibody isotypes. The switching of these isotypes is controlled, to a large extent, by external signals delivered to B cells by CD4^+^ T cells. Therefore, differences in relative levels of vaccine-specific antibody isotypes suggest that environmental exposures may also influence T-cell functions that contribute to the generation and maintenance of optimal responses to vaccination.

Previously in this cohort, we examined early-life PCB exposure in relation to 6-month IgG-specific anti-Haemophilus influenzae type b, tetanus toxoid, and diphtheria toxoid (Jusko et al. 2010) as well as 6-month measures of total IgG, IgA, IgM, and IgE (Jusko et al. 2011). In neither study did we observe associations between maternal or 6-month PCB exposure and specific or total immunoglobulin concentrations. Compared with diphtheria, tetanus, and Haemophilus vaccines, BCG is a live, attenuated vaccine, and because of this it elicits a greater number of effector mechanisms in the immune system (Siegrist 2001). Thus, if immune dysfunction from POP exposure truly exists, then the comprehensive nature of the immune response to BCG increases the likelihood of detecting an association, and may explain the positive findings of the present study compared with our null findings using other antigen-specific outcomes. Similarly, the null results observed for total immunoglobulin concentrations (Jusko et al. 2011) may be explained by their lack of specificity. Total immunoglobulins include all antibodies in an extremely large (> 10^8^) antibody repertoire. Thus, subtle changes involving functional capacity of the immune system may go undetected when nonspecific measures such as total antibody concentrations are used in lieu of more precise and specific assessments (Luster et al. 2005).

The estimated association between 6-month infant PCB and DDE concentrations and anti-BCG antibody levels was relatively large. For instance, we estimated that for both 6-month PCB and 6-month DDE, an IQR increase in concentration was associated with a 37% reduction in anti-BCG IgG antibody levels. As in any observational epidemiological study, the magnitude of these associations raises concerns about the possibility of positive confounding. However, fitting additional 6-month PCB and DDE models that included additional covariate adjustment beyond our prespecified set of covariates suggested that the observed associations are unlikely to be attributable to unmeasured confounding. The relative lack of a change in the crude association following covariate adjustment is a finding that is consistent with previous findings from this cohort (Jusko et al. 2010) and others (Heilmann et al. 2010) and suggests that confounding of the POP–antibody association, empirically evaluated in these populations, is minimal.

Inclusion of duration of exclusive breastfeeding in the 6-month infant serum and PBPK models tended to attenuate the chemical–antibody level associations, because longer breastfeeding was associated with lower BCG antibody levels and higher postnatal PCB and DDE concentrations. Although the finding that longer breastfeeding is associated with a poorer response to BCG vaccination seems paradoxical, a significant literature supports the idea that the relationship between breastfeeding and vaccine responses is complex. For instance, studies have reported enhanced, decreased, or no difference in the immune response to some vaccinations, comparing breastfed to non-breastfed infants (Dórea 2012). For BCG vaccines specifically, one study observed that breastfeeding enhanced lymphocyte blastogenesis (stimulated by purified protein derivative of *Mycobacterium tuberculosis*) (Pabst et al. 1989), and a second study, that examined Mantoux test results at 6 months, found no difference in tuberculin response size across different strata of breastfeeding duration (Goh et al. 1989). Whether breastfeeding is a true confounder or a proxy of exposure that drives its inverse association with anti-BCG antibody levels is difficult to determine. Nevertheless, we observed strong inverse associations between 6-month infant chemical concentrations and BCG-specific antibody levels even with adjustment for duration of exclusive breastfeeding.

Despite the advantages of our study, two limitations deserve mention. First, PCB and DDE concentrations in our population were highly correlated, so fully disentangling their separate associations with anti-BCG antibody levels was not possible. Although the potential immunotoxicity of PCBs and dioxin-like compounds has been studied in human and animals studies (Gascon et al. 2013; Lawrence and Kerkvliet 2006), far fewer studies have examined the potential immunotoxic effects of DDE. Indeed, a recent review of literature regarding POPs and immune outcomes did not identify any human studies evaluating early-life DDE exposure and antibody-specific outcomes (Gascon et al. 2013). The lack of prior vaccine-specific antibody studies of DDE in animals or humans contributes to the uncertainty of whether the DDE–BCG antibody association may be causal, or whether this association is due entirely to the strong positive correlation between PCB and DDE concentrations in this population. Our categorical analysis of high and low concentrations of both compounds suggests that the inverse associations between DDE and anti-BCG antibody levels are not solely the result of correlated exposures.

A second limitation concerns our ability to determine whether the PCB-associated decreases in anti-BCG IgG and IgA levels were attributable to dioxin-like effects on the aryl hydrocarbon receptor (AHR) or mechanisms independent of the AHR. Dioxin-like compounds (including some PCB congeners) bind AHR, which is known to modulate the immune response. However, our method of chemical analysis was predominantly based on high-resolution gas chromatography with electron capture detection, rather than HRMS. As a consequence, many of the values for PCB congeners with dioxin-like activity were below the limit of detection, or co-eluted with other non–dioxin-like PCBs (e.g., PCB-156+171). Although suppression of the immune response following exposure to non–dioxin-like PCBs has been observed in experimental studies in animals (Lyche et al. 2004, 2006), disentangling health effects with highly-correlated PCB congeners in human populations is problematic. Even though PCB-153 (a non–dioxin-like PCB) was the exposure of interest in the present study, PCB-153 concentration among adults in our population has been correlated with the total WHO toxic equivalency (TEQ) (ρ ≥ 0.58), a measure of dioxin-like activity (Van den Berg et al. 2006). Thus, it is difficult to determine whether our PCB-associated findings derive from AHR-dependent or independent mechanisms.

## Conclusions

Although our findings for PCB-153, a common PCB congener observed in most populations around the world (Longnecker et al. 2003), are remarkable, our results for DDE, the main metabolite of DDT, are also noteworthy. The reduced antibody response to BCG with increasing DDE concentrations was observed at concentrations of DDE that are far below those seen in areas where indoor residual spraying is used for malaria vector control (Whitworth et al. 2014). Thus, our findings may have particular implications for areas of the world where DDT is used for malaria vector control, and where, interestingly, rates of tuberculosis are also highest (WHO 2015). In sum, the data presented here provide strong, new evidence that environmental chemicals may lower the response to the vaccine against tuberculosis, a disease responsible for a significant portion of the world’s morbidity and mortality. These data further emphasize that environmental exposures are likely overlooked but critical contributors to the global burden of infectious diseases.

## Supplemental Material

(1.1 MB) PDF
