# Supplementary material for: A Birth Cohort Study of Maternal and Infant Serum PCB-153 and DDE Concentrations and Responses to Infant Tuberculosis Vaccination
Source: Environ Health Perspect. 2015 Dec 9;124(6):813–21. doi: 10.1289/ehp.1510101 (PMC4892928; doi:10.1289/ehp.1510101)
Supplement: (1.1 MB) PDF [file ehp.1510101.s001.acco.pdf]

Environ Health Perspect
DOI: 10.1289/ehp.1510101

Note to readers with disabilities: *EHP* strives to ensure that all journal content is accessible to all readers. However, some figures and Supplemental Material published in *EHP* articles may not conform to 508 standards due to the complexity of the information being presented. If you need assistance accessing journal content, please contact ehp508@niehs.nih.gov. Our staff will work with you to assess and meet your accessibility needs within 3 working days.

Supplemental Material

A Birth Cohort Study of Maternal and Infant Serum PCB-153 and DDE Concentrations and Responses to Infant Tuberculosis Vaccination

Todd A. Jusko, Anneclaire J. De Roos, Sue Y. Lee, Kelly Thevenet-Morrison, Stephen M. Schwartz, Marc-André Verner, Lubica Palkovicova Murinova, Beata Drobná, Anton Kočan, Anna Fabišiková, Kamil Čonka, Tomas Trnovec, Irva Hertz-Picciotto, and B. Paige Lawrence

Table of Contents

Table S1. Measured pre- and postnatal serum PCB and DDE concentrations in relation to 6-month BCG-specific IgG and IgA levels.

Table S2. Serum PCB concentration estimated using a PBPK model in relation to 6-month BCG-specific IgG and IgA levels.

Table S3. Serum DDE concentration estimated using a PBPK model in relation to 6-month BCG-specific IgG and IgA levels.

Table S4. Median 6-month BCG-specific antibody levels by categories of 6-month infant PCB and DDE exposures, with contrasts between each exposure category (n=454).

Figure S1: Unadjusted dose–response relation between quartiles of maternal serum PCB and DDE concentrations and mean 6-month BCG-specific IgG (Panels A and B) and IgA (Panels C and D) levels, across the serum dilution range.

Figure S2: Unadjusted dose–response relation between quartiles of cord serum PCB and DDE concentrations and mean 6-month BCG-specific IgG (Panels A and B) and IgA (Panels C and D) levels, across the serum dilution range.

Figure S3: Unadjusted dose–response relation between quartiles of 6-month infant serum PCB and DDE concentrations and mean 6-month BCG-specific IgG (Panels A and B) and IgA (Panels C and D) levels, across the serum dilution range.

Figure S4: The results from six sensitivity analyses showing the percent change in 6-month BCG-specific IgG level for an interquartile range difference in 6-month infant serum PCB and DDE exposure. The primary model includes adjustment for maternal ethnicity, education, and age, with 6-month PCB and DDE concentration expressed on a lipid basis. The first model is identical to the primary model except that PCB and DDE concentrations are expressed on a ng/ml basis, and 6-month infant lipid concentration is entered as a covariate. The second model adds covariates maternal smoking, parity, district of residence, child sex, and child age at 6-month blood draw. The third model adds a categorical variable for BCG ELISA batch date to the primary model. The fourth model adds an indicator variable to the primary model for high-resolution gas chromatography with electron capture detection versus high-resolution mass spectrometry. The fifth model removes the top and bottom 3% of the respective 6-month infant serum PCB or DDE concentrations. The final sensitivity analysis combines all the previous sensitivity model specifications.

Table S1. Measured pre- and postnatal serum PCB and DDE concentrations in relation to 6-month BCG-specific IgG and IgA levels.

		Percentiles (ng/g lipid)		BCG-Specific IgG			BCG-Specific IgA		
Exposure Measure	N[a]	P25	P75	% difference[b]	95% CI	p-value	% difference[b]	95% CI	p-value
**Maternal PCB**	480	102	234						
Unadjusted				-1.2	(-7.7, 5.8)	0.738	-4.4	(-11.1, 3.0)	0.235
Adjusted[c]				-0.1	(-7.0, 7.3)	0.985	-4.8	(-12.0, 2.9)	0.214
Adjusted + maternal DDE				-0.9	(-8.6, 7.5)	0.836	-5.5	(-13.5, 3.2)	0.206
**Cord PCB**	492	73	188						
Unadjusted				-1.2	(-7.4, 5.5)	0.724	-5.7	(-12.1, 1.2)	0.104
Adjusted[c]				-0.1	(-6.6, 6.8)	0.970	-6.6	(-13.2, 0.4)	0.066
Adjusted + cord DDE				-1.0	(-8.1, 6.6)	0.780	-7.7	(-14.9, 0.2)	0.055
**6-Month Infant PCB**	454	37	248						
Unadjusted				-37.2	(-41.7, -32.3)	<0.001	-33.9	(-39.4, -28.0)	<0.001
Adjusted[d]				-37.1	(-41.7, -32.1)	<0.001	-35.5	(-40.9, -29.6)	<0.001
Adjusted + breastfeeding[e]				-18.9	(-25.6, -11.5)	<0.001	-19.4	(-27.3, -10.6)	<0.001
**Maternal DDE**	480	265	723						
Unadjusted				-2.8	(-8.9, 3.6)	0.384	-3.2	(-9.7, 3.7)	0.354
Adjusted[c]				1.2	(-5.3, 8.1)	0.728	-1.0	(-7.9, 6.4)	0.786
Adjusted + maternal PCB				1.6	(-5.8, 9.5)	0.686	1.5	(-6.5, 10.2)	0.720
**Cord DDE**	492	259	706						
Unadjusted				-2.8	(-8.5, 3.2)	0.346	-2.8	(-9.3, 7.0)	0.387
Adjusted[c]				1.6	(-4.6, 8.1)	0.627	-0.9	(-7.5, 6.1)	0.793
Adjusted + cord PCB				2.0	(-4.8, 9.3)	0.576	2.4	(-5.1, 10.5)	0.543
**6-Month Infant DDE**	454	115	847						
Unadjusted				-36.9	(-41.4, -32.1)	<0.001	-32.0	(-37.6, -25.9)	<0.001
Adjusted[d]				-36.5	(-41.1, -31.5)	<0.001	-33.4	(-39.0, -27.3)	<0.001
Adjusted + breastfeeding[e]				-18.2	(-24.9, -10.9)	<0.001	-15.6	(-23.9, -6.5)	0.001

[a]Number of participants included in each regression model, by exposure. [b]Percent difference in BCG antibody level for a change in the stated interquartile range. [c]Adjusted for maternal ethnicity, education, age, smoking, and parity. [d]Adjusted for maternal ethnicity, education, and age. [e]Adjusted for maternal ethnicity, education, age, and duration of exclusive breastfeeding.

Table S2. Serum PCB concentration estimated using a PBPK model in relation to 6-month BCG-specific IgG and IgA levels.

		Percentiles (ng/g lipid)		BCG-Specific IgG			BCG-Specific IgA		
Exposure Measure	N[a]	P25	P75	% difference[b]	95% CI	p-value	% difference[b]	95% CI	p-value
**Pre- and Postnatal AUC**	510	119	274						
Unadjusted				-8.6	(-14.4, -2.4)	0.007	-11.1	(-17.2, -4.6)	0.001
Adjusted[c]				-7.7	(-13.7, -1.2)	0.020	-11.8	(-18.1, -5.1)	<0.001
Adjusted + breastfeeding[d]				-3.1	(-8.4, 2.4)	0.264	-8.3	(-14.2, -2.0)	0.011
**Pre- and Postnatal Peak**	510	187	427						
Unadjusted				-10.6	(-16.3, -4.6)	<0.001	-11.9	(-17.9, -5.5)	<0.001
Adjusted[c]				-9.3	(-15.2, -3.1)	0.004	-12.3	(-18.5, -5.7)	<0.001
Adjusted + breastfeeding[d]				-3.3	(-8.6, 2.3)	0.236	-7.5	(-13.5, -1.1)	0.022
**Postnatal Month 1**	517	152	350						
Unadjusted				-1.4	(-7.6, 5.3)	0.677	-5.2	(-11.7, 1.7)	0.134
Adjusted[e]				1.9	(-4.6, 8.9)	0.572	-3.0	(-9.9, 4.3)	0.406
Adjusted + breastfeeding[f]				2.1	(-4.4, 9.0)	0.542	-2.9	(-9.7, 4.4)	0.421
**Postnatal Month 2**	517	140	354						
Unadjusted				-9.0	(-15.1, -2.4)	0.008	-12.3	(-18.6, -5.4)	<0.001
Adjusted[e]				-6.3	(-12.7, 0.7)	0.075	-10.8	(-17.5, -3.5)	0.004
Adjusted + breastfeeding[f]				0.0	(-6.6, 7)	0.995	-5.1	(-12, 2.4)	0.178
**Postnatal Month 3**	517	124	327						
Unadjusted				-16.1	(-21.6, -10.3)	<0.001	-17.8	(-23.6, -11.5)	<0.001
Adjusted[e]				-14.2	(-19.9, -8.1)	<0.001	-17.0	(-23.1, -10.5)	<0.001
Adjusted + breastfeeding[f]				-1.9	(-8.3, 4.9)	0.569	-7.0	(-13.8, 0.4)	0.064
**Postnatal Month 4**	517	112	316						
Unadjusted				-21.3	(-26.3, -15.9)	<0.001	-21.6	(-27.1, -15.7)	<0.001
Adjusted[e]				-19.8	(-25.1, -14.2)	<0.001	-21.3	(-27, -15.2)	<0.001
Adjusted + breastfeeding[f]				-4.3	(-10.5, 2.4)	0.204	-8.7	(-15.6, -1.3)	0.022
**Postnatal Month 5**	517	103	316						
Unadjusted				-25.4	(-30.2, -20.2)	<0.001	-24.5	(-29.9, -18.7)	<0.001
Adjusted[e]				-24.2	(-29.2, -18.7)	<0.001	-24.5	(-30, -18.4)	<0.001
Adjusted + breastfeeding[f]				-6.8	(-12.9, -0.2)	0.044	-10.4	(-17.4, -2.9)	0.008
**Postnatal Month 6**	517	96	312						
Unadjusted				-27.7	(-32.4, -22.6)	<0.001	-26.1	(-31.4, -20.3)	<0.001
Adjusted[e]				-26.7	(-31.6, -21.4)	<0.001	-26.3	(-31.8, -20.3)	<0.001
Adjusted + breastfeeding[f]				-8.9	(-15, -2.4)	0.008	-11.9	(-18.9, -4.3)	0.003
**Postnatal AUC**	517	126	321						
Unadjusted				-16.0	(-21.5, -10.1)	<0.001	-17.4	(-23.2, -11.1)	<0.001
Adjusted[e]				-13.9	(-19.7, -7.7)	<0.001	-16.5	(-22.6, -9.9)	<0.001
Adjusted + breastfeeding[f]				-4.2	(-9.8, 1.6)	0.152	-8.6	(-14.9, -1.9)	0.013
**Postnatal Peak**	517	179	419						
Unadjusted				-10.2	(-16, -4.1)	0.002	-12.2	(-18.3, -5.7)	<0.001
Adjusted[e]				-7.3	(-13.4, -0.7)	0.030	-10.5	(-17, -3.6)	0.004
Adjusted + breastfeeding[f]				-3.5	(-8.8, 2.1)	0.216	-7.4	(-13.4, -1.0)	0.025

[a]Number of participants included in each regression model, by exposure. [b]Percent difference in BCG antibody level for a change in the stated interquartile range. [c]Adjusted for maternal ethnicity, education, age, smoking, and parity. [d]Adjusted for maternal ethnicity, education, age, smoking, parity, and duration of exclusive breastfeeding. [e]Adjusted for maternal ethnicity, education, and age. [f]Adjusted for maternal ethnicity, education, age, and duration of exclusive breastfeeding

Table S3. Serum DDE concentration estimated using a PBPK model in relation to 6-month BCG-specific IgG and IgA levels.

		Percentiles (ng/g lipid)		BCG-Specific IgG			BCG-Specific IgA		
Exposure Measure	N[a]	P25	P75	% difference[2]	95% CI	p-value	% difference[b]	95% CI	p-value
**Pre- and Postnatal AUC**	510	321	872						
Unadjusted				-7.4	(-13, -1.5)	0.014	-7.1	(-13.1, -0.7)	0.031
Adjusted[c]				-3.6	(-9.6, 2.7)	0.254	-5.2	(-11.6, 1.6)	0.132
Adjusted + breastfeeding[d]				-0.6	(-5.7, 4.8)	0.837	-2.7	(-8.7, 3.7)	0.397
**Pre- and Postnatal Peak**	510	506	1345						
Unadjusted				-8.2	(-13.6, -2.6)	0.005	-7.1	(-12.9, -0.8)	0.027
Adjusted[c]				-4.4	(-10.1, 1.8)	0.159	-5.0	(-11.2, 1.7)	0.141
Adjusted + breastfeeding[d]				-0.6	(-5.5, 4.7)	0.832	-1.8	(-7.7, 4.4)	0.559
**Postnatal Month 1**	517	398	1099						
Unadjusted				-2.3	(-8.2, 4.0)	0.458	-2.5	(-8.9, 4.4)	0.469
Adjusted[e]				2.4	(-4, 9.1)	0.473	0.9	(-6.0, 8.3)	0.806
Adjusted + breastfeeding[f]				2.1	(-4.1, 8.8)	0.512	0.7	(-6.1, 8.0)	0.852
**Postnatal Month 2**	517	382	1065						
Unadjusted				-7.5	(-12.9, -1.6)	0.013	-7.2	(-13.2, -0.9)	0.026
Adjusted[e]				-3.6	(-9.5, 2.6)	0.248	-4.8	(-11.2, 2.0)	0.164
Adjusted + breastfeeding[f]				0.5	(-5.3, 6.7)	0.865	-0.7	(-7.1, 6.1)	0.826
**Postnatal Month 3**	517	341	1030						
Unadjusted				-13.4	(-18.6, -7.8)	<0.001	-12.3	(-18.1, -6.1)	<0.001
Adjusted[e]				-10.3	(-15.9, -4.3)	0.001	-10.5	(-16.7, -3.9)	0.002
Adjusted + breastfeeding[f]				-1.4	(-7.2, 4.7)	0.636	-2.7	(-9.2, 4.3)	0.444
**Postnatal Month 4**	517	318	1010						
Unadjusted				-17.6	(-22.5, -12.3)	<0.001	-15.6	(-21.1, -9.7)	<0.001
Adjusted[e]				-15.0	(-20.3, -9.4)	<0.001	-14.4	(-20.2, -8.1)	<0.001
Adjusted + breastfeeding[f]				-2.9	(-8.6, 3.1)	0.329	-3.7	(-10.2, 3.2)	0.286
**Postnatal Month 5**	517	290	1004						
Unadjusted				-21.2	(-26.0, -16.0)	<0.001	-18.3	(-23.8, -12.4)	<0.001
Adjusted[e]				-19.0	(-24.1, -13.4)	<0.001	-17.4	(-23.3, -11.1)	<0.001
Adjusted + breastfeeding[f]				-4.4	(-10.1, 1.7)	0.155	-4.6	(-11.3, 2.6)	0.205
**Postnatal Month 6**	517	264	997						
Unadjusted				-23.8	(-28.6, -18.6)	<0.001	-20.3	(-25.9, -14.3)	<0.001
Adjusted[e]				-21.8	(-27.0, -16.2)	<0.001	-19.6	(-25.6, -13.2)	<0.001
Adjusted + breastfeeding[f]				-5.7	(-11.5, 0.6)	0.076	-5.5	(-12.5, 2.1)	0.150
**Postnatal AUC**	517	356	1005						
Unadjusted				-12.7	(-17.8, -7.3)	<0.001	-11.3	(-16.9, -5.2)	<0.001
Adjusted[e]				-9.5	(-15.0, -3.6)	0.002	-9.4	(-15.5, -2.9)	0.005
Adjusted + breastfeeding[f]				-1.9	(-7.0, 3.4)	0.467	-2.9	(-8.9, 3.4)	0.359
**Postnatal Peak**	517	497	1345						
Unadjusted				-8.5	(-13.9, -2.8)	0.004	-7.4	(-13.3, -1.1)	0.022
Adjusted[e]				-4.5	(-10.3, 1.7)	0.154	-4.7	(-11.1, 2.1)	0.171
Adjusted + breastfeeding[f]				-1.4	(-6.4, 3.8)	0.584	-2.1	(-8.0, 4.2)	0.503

[a]Number of participants included in each regression model, by exposure. [b]Percent difference in BCG antibody level for a change in the stated interquartile range. [c]Adjusted for maternal ethnicity, education, age, smoking, and parity. [d]Adjusted for maternal ethnicity, education, age, smoking, parity, and duration of exclusive breastfeeding. [e]Adjusted for maternal ethnicity, education, and age. [f]Adjusted for maternal ethnicity, education, age, and duration of exclusive breastfeeding

Table S4. Median 6-month BCG-specific antibody levels by categories of 6-month infant PCB and DDE exposures, with contrasts between each exposure category (n=454).

	BCG-Specific IgG						BCG-Specific IgA					
			Exposure Category Contrasts						Exposure Category Contrasts			
Exposure Category PCB/DDE[a]	Median	95% CI	Contrast	% Difference	95% CI	p-value	Median	95% CI	Contrast	% Difference	95% CI	p-value
No adjustment for duration of exclusive breastfeeding[b]												
1: Low/Low	0.74	0.66, 0.83	1 vs 2	11%	-8, 34	0.298	0.34	0.30, 0.39	1 vs 2	15%	-8, 42	0.223
2: Low/High	0.67	0.55, 0.81	1 vs 3	36%	13, 65	0.001	0.30	0.24, 0.37	1 vs 3	70%	37, 111	<0.001
3: High/Low	0.54	0.45, 0.66	1 vs 4	94%	74, 116	<0.001	0.20	0.16, 0.25	1 vs 4	85%	64, 109	<0.001
4: High/High	0.38	0.35, 0.42	2 vs 3	23%	-4, 57	0.100	0.18	0.16, 0.21	2 vs 3	48%	12, 97	0.006
	Interaction p=0.012[c]		2 vs 4	75%	45, 112	<0.001	Interaction p=0.352		2 vs 4	62%	30, 101	<0.001
			3 vs 4	42%	18, 72	<0.001			3 vs 4	9%	-12, 35	0.432
Adjustment for duration of exclusive breastfeeding[d]												
1: Low/Low	0.63	0.57, 0.71	1 vs 2	3%	-14, 22	0.751	0.29	0.26, 0.34	1 vs 2	7%	-13, 32	0.522
2: Low/High	0.62	0.52, 0.74	1 vs 3	11%	-8, 32	0.270	0.28	0.22, 0.34	1 vs 3	40%	13, 74	0.002
3: High/Low	0.57	0.48, 0.68	1 vs 4	36%	20, 54	<0.001	0.21	0.17, 0.26	1 vs 4	34%	16, 56	<0.001
4: High/High	0.47	0.42, 0.52	2 vs 3	8%	-14, 35	0.530	0.22	0.19, 0.25	2 vs 3	31%	0, 72	0.049
	Interaction p=0.066		2 vs 4	32%	10, 59	0.003	Interaction p=0.406		2 vs 4	25%	0, 56	0.043
			3 vs 4	23%	3, 47	0.021			3 vs 4	-4%	-23, 18	0.672

[a]Categories defined by median splits for 6-month infant PCB (113 ng/g lipid) and DDE (388 ng/g lipid) concentrations. [b]Adjusted for maternal ethnicity, education, and age. [c]P-value for the interaction between 6-month PCB and DDE. [d]Adjusted for maternal ethnicity, education, age, and duration of exclusive breastfeeding.

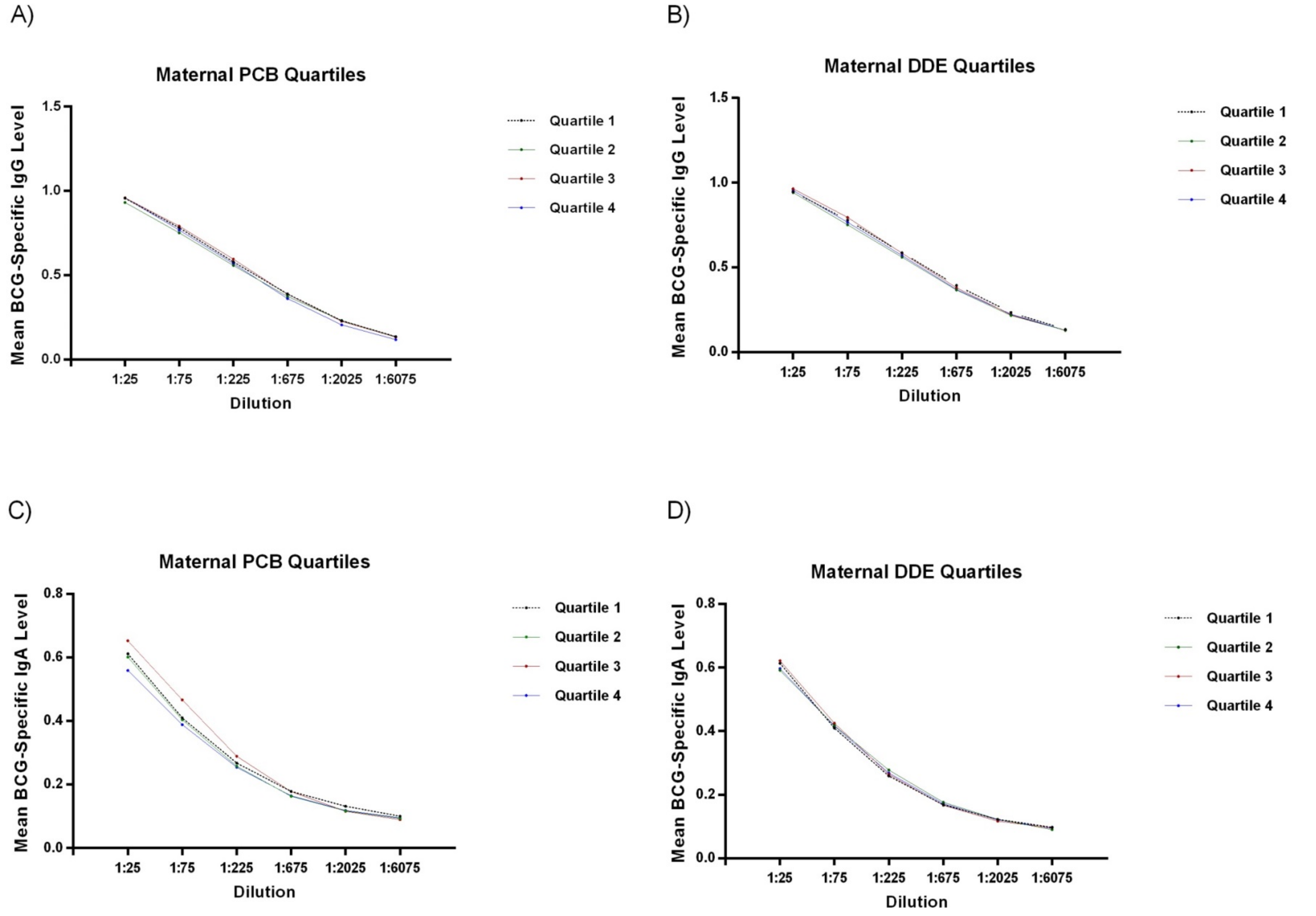

Figure S1: Unadjusted dose–response relation between quartiles of maternal serum PCB and DDE concentrations and mean 6-month BCG-specific IgG (Panels A and B) and IgA (Panels C and D) levels, across the serum dilution range.

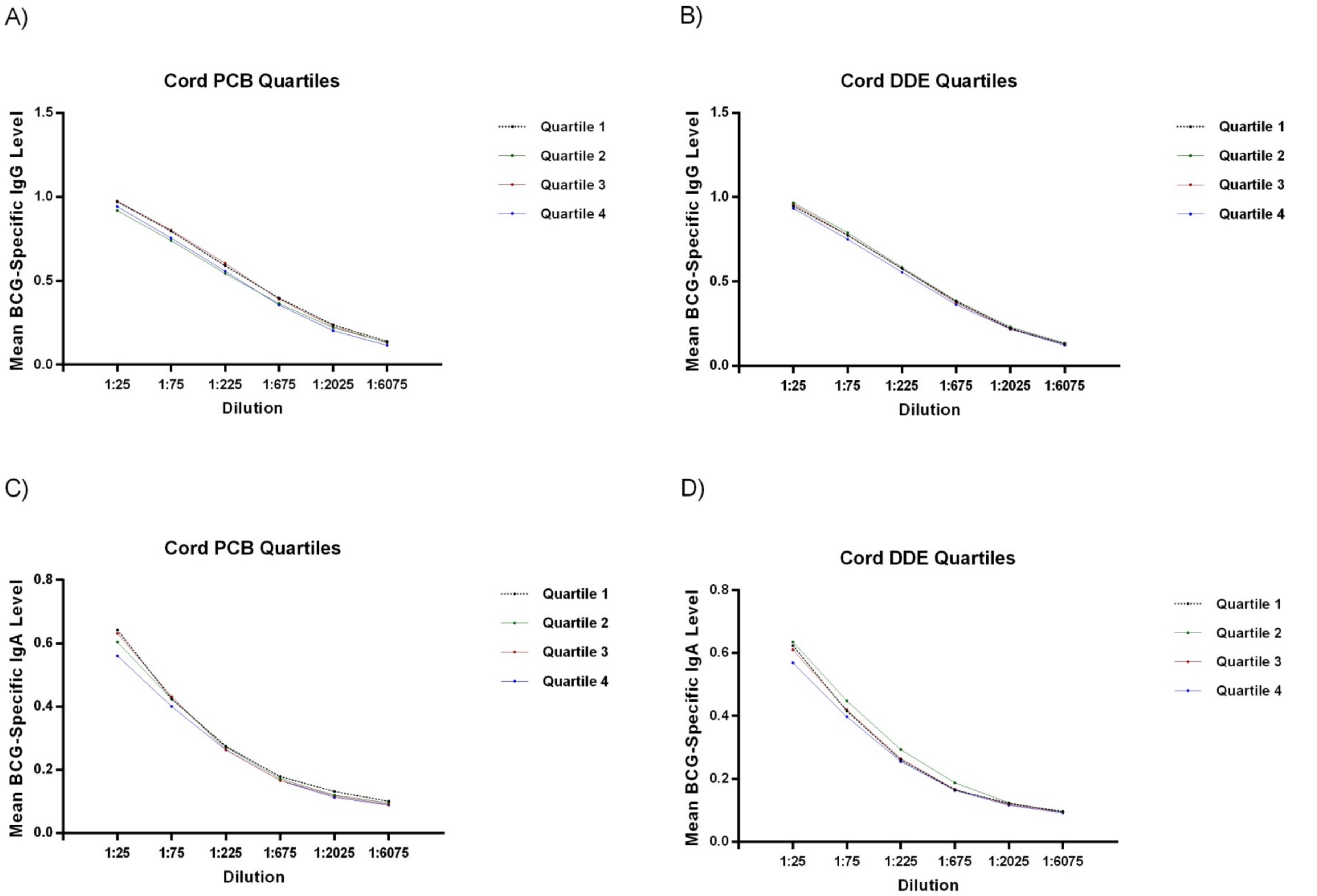

Figure S2: Unadjusted dose–response relation between quartiles of cord serum PCB and DDE concentrations and mean 6-month BCG-specific IgG (Panels A and B) and IgA (Panels C and D) levels, across the serum dilution range.

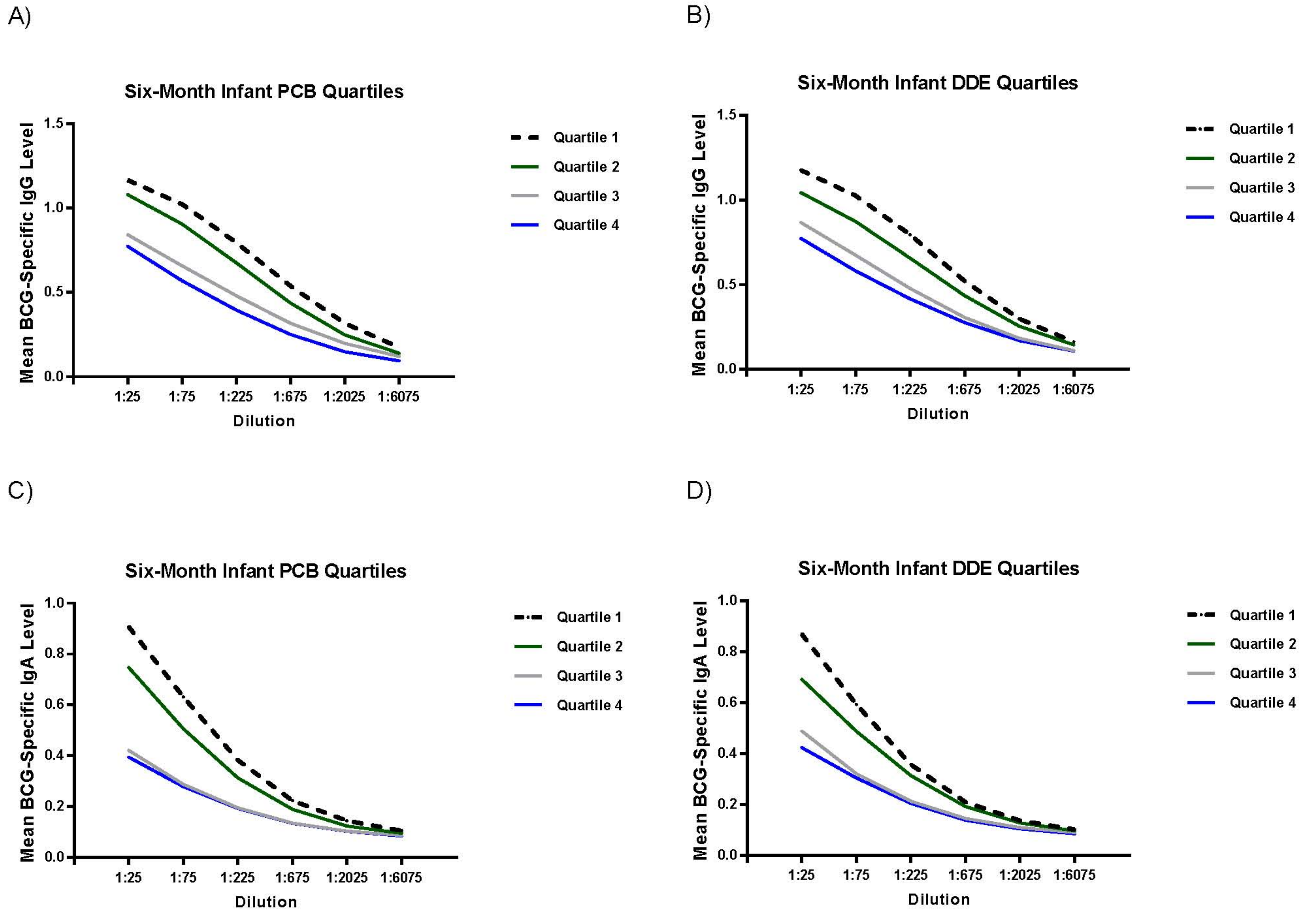

Figure S3: Unadjusted dose–response relation between quartiles of 6-month infant serum PCB and DDE concentrations and mean 6-month BCG-specific IgG (Panels A and B) and IgA (Panels C and D) levels, across the serum dilution range.

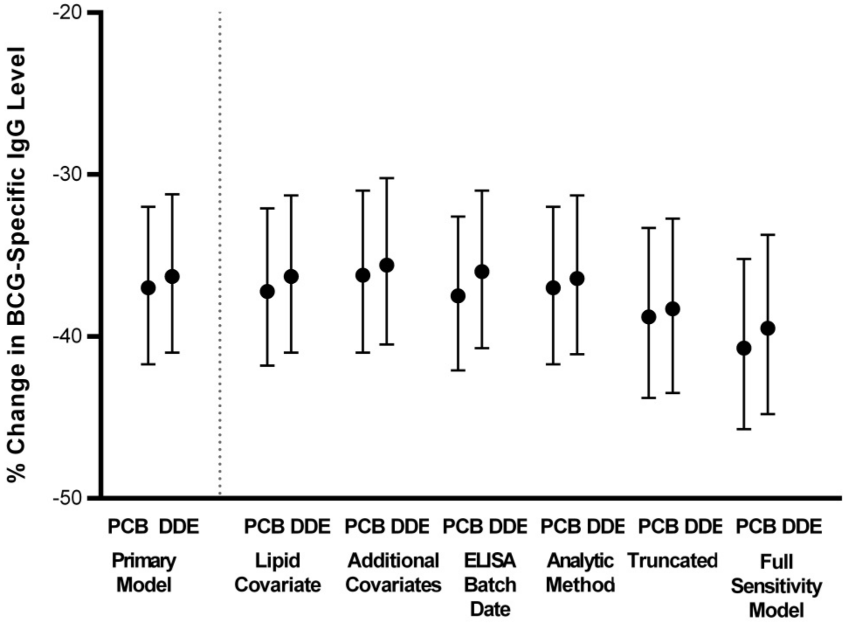

Figure S4: The results from six sensitivity analyses showing the percent change in 6-month BCG-specific IgG level for an interquartile range difference in 6-month infant serum PCB and DDE exposure. The primary model includes adjustment for maternal ethnicity, education, and age, with 6-month PCB and DDE concentration expressed on a lipid basis. The first model is identical to the primary model except that PCB and DDE concentrations are expressed on a ng/ml basis, and 6-month infant lipid concentration is entered as a covariate. The second model adds covariates maternal smoking, parity, district of residence, child sex, and child age at 6-month blood draw. The third model adds a categorical variable for BCG ELISA batch date to the primary model. The fourth model adds an indicator variable to the primary model for high-resolution gas chromatography with electron capture detection versus high-resolution mass spectrometry. The fifth model removes the top and bottom 3% of the respective 6-month infant serum PCB or DDE concentrations. The final sensitivity analysis combines all the previous sensitivity model specifications.
